# Advances in Diagnostic Modalities for *Helicobacter pylori* Infection

**DOI:** 10.3390/life14091170

**Published:** 2024-09-17

**Authors:** Haider Ghazanfar, Nismat Javed, Raul Reina, Ornela Thartori, Ali Ghazanfar, Harish Patel

**Affiliations:** 1 Division of Gastroenterology, BronxCare Health System, Bronx, NY 10457, USA; 2 BronxCare Health System, Bronx, NY 10457, USA; 3 Fauji Foundation Hospital, Rawalpindi 45000, Pakistan

**Keywords:** *Helicobacter pylori*, diagnostic modalities, advances, NGS, MALDI-TOF MS

## Abstract

*Helicobacter pylori* (*H. pylori*) infection is a widespread global health issue with a varying prevalence influenced by geography, socioeconomic status, and demographics. In the U.S., the prevalence is lower, though certain groups, such as older adults and immigrants from high-prevalence regions, show higher rates. The decrease in infection rates in developed countries is due to improved sanitation, antibiotics, and healthcare, whereas developing countries continue to experience high rates due to poor living conditions. *H. pylori* infection can be asymptomatic or cause symptoms like dyspepsia, abdominal pain, bloating, nausea, and loss of appetite. Pathophysiologically, *H. pylori* contribute to conditions such as gastritis, peptic ulcers, and gastric cancer through mechanisms including urease production and the release of virulence factors, leading to chronic inflammation and an increased cancer risk. Diagnostic methods for *H. pylori* have progressed significantly. Non-invasive techniques, such as serological assays, stool antigen tests, and urea breath tests, are practical and sensitive. Invasive methods, including endoscopic biopsy and molecular diagnostics, are more definitive but resource intensive. Recent advancements in diagnostic technology, including matrix-assisted laser desorption/ionization time-of-flight mass spectrometry (MALDI-TOF MS), biosensor technology, and next-generation sequencing (NGS), promise improved speed, accuracy, and accessibility. These innovations are expected to enhance the detection and management of *H. pylori*, potentially reducing the global disease burden. This review aims to discuss these diagnostic modalities with a focus on further advances under investigation.

## 1. Introduction

*H. pylori* infection remains a significant global health concern, with varying prevalence and incidence rates across countries, geography, ethnicity, age, and socioeconomic factors. It is among the most widespread chronic bacterial infections globally [1], and it poses significant public health challenges, especially in developing nations. In developing countries, the incidence can be as high as 70–90%, whereas in developed countries, it ranges from 30 to 50%. The prevalence of *H. pylori* infection in the United States is lower than global averages, with estimates ranging from 20 to 30%. However, the prevalence can be higher in specific subpopulations, such as older adults, immigrants from high-prevalence regions, and individuals with lower socioeconomic status. Over recent years, there has been a notable decline in the prevalence of *H. pylori* infection in many developed countries, including the U.S., likely due to improved sanitation, the widespread use of antibiotics, and better overall healthcare. In contrast, developing countries experience high prevalence rates due to poorer sanitation and living conditions [2].

### 1.1. Clinical Signs and Symptoms

Many individuals can be asymptomatic with *H. pylori* infection. Still, when symptoms do occur, they often include dyspepsia, abdominal pain that is usually described as a burning or gnawing sensation, bloating, nausea and vomiting, and loss of appetite [1]. Many patients also present with atypical chest pain that can represent atherosclerotic disease in patients with *H. pylori* infection. It has been explained that treating gastrointestinal symptoms in some patients has improved the rate of atherogenesis [3]. 

### 1.2. Pathophysiology

A Gram-negative bacterium that resides in the human stomach, *H. pylori,* causes various gastrointestinal conditions, such as gastritis, peptic ulcers, and gastric cancer. *H. pylori* attaches to gastric epithelial cells using various adhesins. This attachment is essential for colonization and survival in the stomach’s acidic environment. *H. pylori* produces urease, which breaks down urea into ammonia, neutralizing the stomach acid, creating a more hospitable environment for the bacterium, and protecting it from acidic conditions. There are a few toxins associated with *H. pylori*. The cytotoxin-associated gene A (CagA) protein is secreted into cells via a type IV secretion system [4]. Inside the host cells, it becomes phosphorylated and interacts with several cellular pathways, causing changes in cell signaling, inflammation, and an increased risk of cancer. **Vacuolating cytotoxin A** (VacA) induces vacuole formation in epithelial cells, disrupts mitochondrial function, triggers apoptosis, and affects immune responses [5]. This versatile toxin helps the bacterium evade the host’s immune defenses. *H. pylori* infection triggers a significant inflammatory response in the gastric mucosa. The bacteria and their products stimulate the production of pro-inflammatory cytokines like IL-8, attracting neutrophils and other immune cells to the infection site. Chronic inflammation can result in tissue damage, ulcers, and a higher risk of gastric cancer [6]. *H. pylori* employs several strategies to evade the host immune system, including phase variation [7], modification of lipopolysaccharides, and secretion of immunomodulatory factors. *H. pylori* can alter gastric acid secretion. In some cases, it increases acid production, leading to duodenal ulcers. In other cases, especially with strains causing strong inflammation, it can cause atrophic gastritis and reduced acid production, increasing the risk of gastric cancer [8]. The virulence of *H. pylori* strains varies, with certain genetic markers (such as cagA, vacA, and iceA) associated with more severe disease outcomes. Host genetic factors also contribute to susceptibility and disease severity.

### 1.3. Complications

Understanding the clinical signs and ensuring timely diagnosis and treatment are crucial for preventing these adverse outcomes and complications, such as duodenal or gastric ulcers, which can bleed or perforate. *H. pylori* is a significant risk factor for gastric adenocarcinoma [9]. Chronic inflammation from the infection can lead to changes in the stomach lining, increasing cancer risk. *H. pylori* infection is associated with mucosa-associated lymphoid tissue (MALT) lymphoma, a rare cancer affecting the stomach lining. However, the presence of *H. pylori* infection has been mentioned as a protective risk factor for esophageal adenocarcinoma in few cases [10]. Persistent infection can cause chronic gastritis, leading to ongoing stomach pain and discomfort. *H. pylori* infection can contribute to functional dyspepsia, causing long-term digestive discomfort without visible ulcers [11].

### 1.4. Management

The treatment for *H. pylori* has evolved, and management has evolved to multiple lines of therapy. However, resistance to antibiotics, particularly clarithromycin, has become a significant challenge. The most common treatment now involves a combination of a PPI and two antibiotics, typically administered for 7 to 14 days [12]. Success rates are generally high, around 90%, but factors like patient compliance and antibiotic resistance can affect outcomes [12]. In cases where the initial treatment fails, second-line therapies or quadruple therapy may be necessary. In regions with high antibiotic resistance, quadruple therapy is preferred, and the treatment’s success is confirmed through tests, such as the urea breath test or fecal antigen test [12]. Additionally, probiotics have been shown to aid in eradicating *H. pylori* by promoting gut health, secreting antimicrobial substances, and reducing side effects from antibiotic therapy [12]. 

## 2. Non-Invasive Diagnostic Modalities

**Figure 1:**
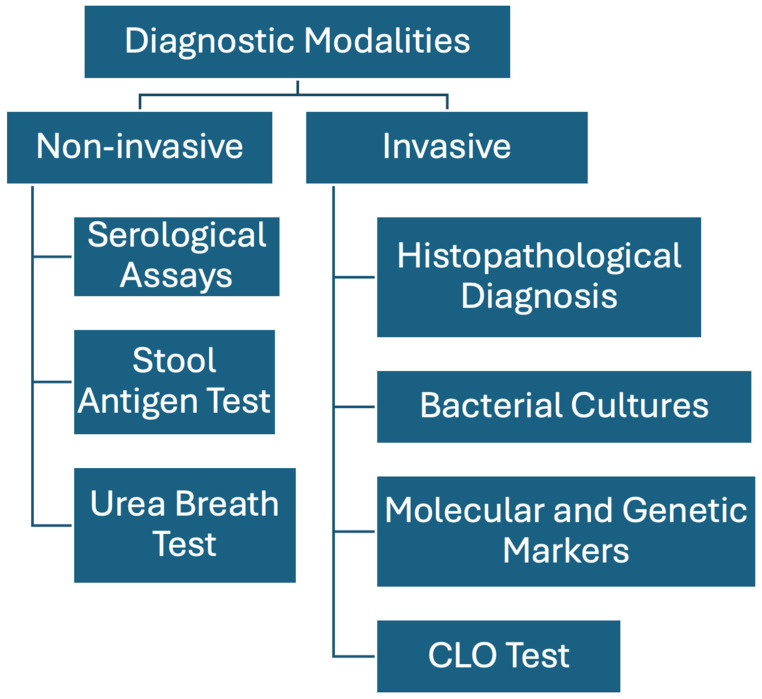
Summary of diagnostic tests.

Various diagnostic methods, both invasive and non-invasive, are available for detecting *H. pylori* infection (Figure 1) [13,14,15]. Invasive techniques typically involve a biopsy followed by cultures and histological examination of mucosal samples. These invasive tests, while specific, rely heavily on the precision of the biopsy process. Additionally, they are time-consuming and require specialized laboratory resources, making them less accessible.

Due to these challenges, non-invasive diagnostic methods have been developed and are now widely used. These non-invasive methods fall into three primary categories: serological assays, stool antigen tests, or urea breath tests. These non-invasive techniques are generally easier to perform and do not require the specialized facilities needed for invasive procedures.

### 2.1. Serological Assay

Serological testing involves measuring specific antibodies against *H. pylori* or its toxins in serum samples. This diagnostic method is advantageous because it is inexpensive and provides results quickly [16]. Individuals infected with *H. pylori* typically produce specific circulating antibodies, including IgG, IgA, and IgM, which can be detected using serological tests. Most commercially available tests focus on detecting IgG antibodies.

Infections with CagA positive strains (type I) are often associated with more severe gastroduodenal diseases. Therefore, detecting a serological response to the CagA antigen can offer clinically valuable insights into the infecting strain. Various techniques, such as enzyme-linked immunosorbent assay (ELISA), latex agglutination, and immunochromatography, are available for detecting serum antibodies [17].

Rapid tests are also available, suitable for point-of-care use and rely on either latex agglutination or immunochromatographic technology. 

The latex agglutination test, for example, is a quick method that detects antibodies to *H. pylori* in a patient’s serum by using latex particles coated with an antigen extracted from the bacterium. If *H. pylori* is present, it will react with the latex particles, causing visible clumping. Although the interpretation of this test can be subjective, it remains widely used due to its simplicity and the fact that it provides results within minutes compared to the hour or more required for ELISA tests [16]. Immunochromatographic tests allow the sample to flow through an absorbent device, where an anti-human immunoglobulin dye conjugate binds to human IgG antibodies if present, creating a visible colored band [16].

The performance of serological assays has been found to have an overall sensitivity and specificity of 85% and 79%, respectively, with no significant differences between various assays [18]. However, serological tests are less effective for monitoring treatment response because patients may retain *H. pylori*-specific antibodies in their serum for several months after eradication. Qualitative tests can remain positive for up to 3 years after successful treatment, while quantitative assays may not show significant declines for 6 to 12 months [17]. Additionally, false-positive serology results are more common in populations with low prevalence, as the positive predictive value of antibody testing is heavily influenced by the prevalence of *H. pylori* infection in the area. Although the modality has its advantages due to widespread availability at a lower cost, the limitations include reduced sensitivity and specificity for monitoring treatment for eradication [17,18]. Therefore, if serological results are positive, confirmation with more reliable diagnostic methods, whether invasive or non-invasive, is necessary.

### 2.2. Stool Antigen Test

The *H. pylori* stool antigen (HpSA) test offers a distinct advantage over other commonly used diagnostic methods by detecting the presence of the bacterium itself rather than antibodies produced in response to it. This allows for the identification of an active, ongoing infection. In contrast, serological tests can only indicate whether a person has been in contact with the bacterium at some point without distinguishing between current and past infections. In addition to being non-invasive and cost-effective, the HpSA test is noted for its good sensitivity, specificity, and overall reliability. This makes it a valuable tool not only for diagnosing *H. pylori* infection but also for monitoring the effectiveness of treatment [16]. The HpSA test is available in two formats: the traditional ELISA and the more rapid version.

#### 2.2.1. HpSA ELISA Test

According to various studies and the International Consensus Report, both the urea breath test (UBT) and stool antigen test are regarded as first-line diagnostic methods, with sensitivity and specificity exceeding 90%. For diagnosing *H. pylori* infection, the HpSA ELISA test demonstrates sensitivity and specificity values of 93.3% and 93.2% [19,20,21], respectively, which are only slightly lower than those of the UBT. However, it is important to emphasize that the accuracy of the HpSA test is heavily dependent on the correct collection and storage of samples. Mishandling can result in a significant drop in sensitivity, potentially as low as 69%, underscoring the need for careful management of the testing process and therefore, being a potential limitation. 

#### 2.2.2. Rapid HpSA Test

The rapid HpSA test is a user-friendly diagnostic tool that employs immunochromatographic technology to detect *H. pylori* antigens in human feces. It has a sensitivity of 91.3% and a specificity of 93.5%, making it an effective method for diagnosing *H. pylori* infection [22]. This test is particularly beneficial for patients who may have difficulty performing a urea breath test, such as young children or elderly individuals. Additionally, the rapid HpSA test is not only useful for initial diagnosis but also for monitoring the success of eradication therapy, offering a practical and accessible option for various patient populations. 

### 2.3. Urea Breath Test (UBT)

The urea breath test (UBT) is another widely available diagnostic tool with high sensitivity and specificity, typically ranging from 90% to 100%. The test is straightforward to administer, but it requires patient cooperation, which can be challenging in certain groups, such as very young children or elderly patients [23]. The UBT works by taking advantage of the *H. pylori* bacterium’s ability to produce the enzyme urease. This enzyme breaks down urea in the stomach, releasing carbon dioxide and ammonia, which neutralize gastric acid and create a more favorable environment for the bacterium. The test involves administering urea labeled with a carbon isotope (either 13C or 14C), which, once ingested, is broken down by the urease. The resulting carbon dioxide is absorbed through the gastric mucosa into the bloodstream, eventually reaching the lungs, where it is exhaled and measured, providing a clear indication of the presence of *H. pylori* infection. While the test is an important measure for diagnostic accuracy, the baseline level of urease in oral flora can vary on a spectrum depending on the patient and can impact results [24]. 

## 3. Invasive Diagnostic Modalities

These diagnostic modalities include collecting mucosal specimens through endoscopic biopsy, histological examination, bacterial cultures, a rapid urease test, and PCR-based analyses [25]. Esophagogastroduodenoscopy (EGD) is the most reliable and recommended diagnostic procedure for infection [26].

### 3.1. Histopathological Diagnosis

A histological examination of biopsy samples can provide critical insights into the extent of inflammation and related conditions, such as gastric cancer, intestinal metaplasia, and gastritis [27]. The commonly used staining technique involves hematoxylin and eosin (H and E), though other stains like Giemsa, Genta stain, or Warthin–Starry may also be utilized. Immunohistochemistry is sometimes necessary for a precise histological diagnosis, especially when H and E staining does not yield sufficient information, such as in cases where the bacterial load in the sample is low [27,28]. The accuracy of a histologic diagnosis can be influenced by several factors, including the biopsy’s location, size, and number of samples taken. To optimize detection, the Sydney system recommends obtaining biopsy specimens from five different sites, including the pylorus, antrum, corpus, and angulus [29]. Histological examination is considered the most reliable diagnostic method and remains the preferred approach for confirming suspected *H. pylori* infections [30].

### 3.2. Bacterial Culture

Based on Koch’s postulates, microbiological examination of cultures obtained from mucosal samples after an endoscopy is considered a reliable method for detecting and diagnosing *H. pylori* infection. However, this approach has several limitations, such as the bacterium’s fastidious nature, the necessity for specific growth conditions and specialized microbiological equipment, and the often-low recovery rates of the bacterium from infected samples [31]. Despite these challenges, bacterial culture remains the only method that allows for detailed analysis of the bacterium’s characteristics, including the strain type and its antibiotic sensitivities [32]. This makes it an indispensable tool, especially in cases where antibiotic resistance is a concern, as it can guide targeted treatment strategies.

### 3.3. Molecular and Genetic Markers

Advances in molecular biology have led to the development of highly accurate diagnostic tools for detecting *H. pylori* infection. These molecular methods, such as reverse transcription polymerase chain reaction (RT-PCR), are now preferred over traditional techniques due to their reliability and precision. RT-PCR, in particular, not only enables the detection of *H. pylori* but also facilitates the characterization of potential antibiotic resistance, which is crucial for effective treatment [33]. This molecular approach relies on specific biomolecules or markers that help in the early diagnosis of *H. pylori* infection. These markers include 16S rRNA, 16S rDNA, and the virulence factors CagA and VacA, among others [29]. Recent research has highlighted the effectiveness of molecular markers like 16S rRNA in accurately detecting *H. pylori*, especially in cases where traditional methods might yield negative results despite the presence of the pathogen [34]. Moreover, innovative techniques, such as the RPA-CRISPR-Cas12a-based method, have been proposed for *H. pylori* detection. These novel methods offer exceptionally high sensitivity, with the ability to detect the bacterium at concentrations as low as 2 ng/μL, making them promising tools for use in various clinical settings [35].

### 3.4. CLO Test

The Campylobacter-like organism (CLO) test depends on the organism’s production of urease. The release of ammonia increases the test medium’s pH and changes the indicator’s color. The change in color indicates that biopsies are positive for *H. pylori* [36]. Previous evidence has shown that the CLO test has low sensitivity compared to other noninvasive methods such as HpSA. Therefore, when *H. pylori* status needs to be confirmed, using other diagnostic modalities along with the CLO test would be a suitable method for diagnosis [37]. On the other hand, recent research has revealed a high sensitivity of 100% and sensitivity of 50–85% for the CLO test when assessing for *H. pylori* infection and a significant association with esophageal ulcers (*p*-value = 0.01) [38].

## 4. Advances in Diagnostic Modalities

**Figure 2:**
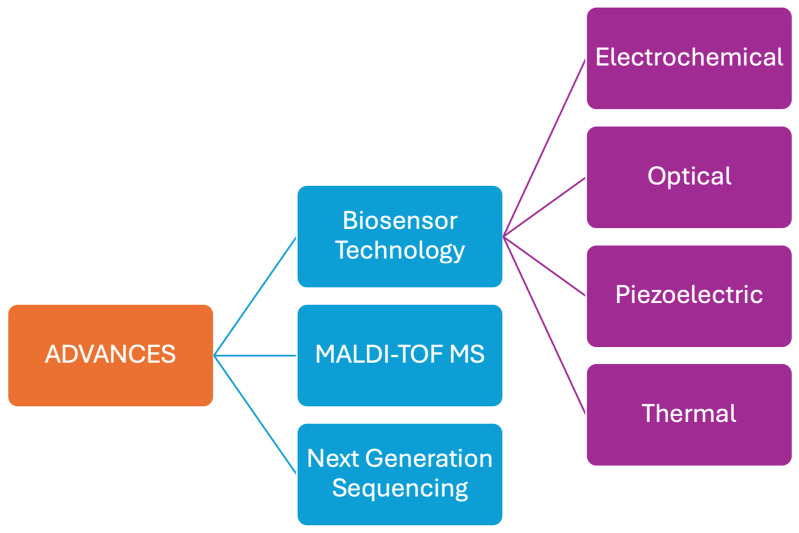
Advances in diagnostic modalities.

Recognition of *H. pylori* as the leading player in several gastroduodenal diseases has contributed to the growing demand for its diagnosis. It possesses a high genomic diversity, which is one of the reasons for the persistent infection in the stomach [9]. The gold standard for a bacterial infection is to culture the species, but not in the case of *H. pylori*. The diagnostic approach to *H. pylori* infection involves non-invasive and invasive methods, each with advantages and limitations [39]. Recent advances in *H. pylori* detection have focused on improving non-invasive methods, enhancing molecular techniques, and utilizing novel technologies. Molecular diagnostics, like PCR and NGS, offer detailed genetic insights and high accuracy. Immunoassays and breath tests provide non-invasive options, while biosensors and point-of-care devices represent the cutting-edge in rapid and accessible diagnostics. These advancements promise to improve the management of *H. pylori* infections and reduce the associated disease burden (Figure 2).

### 4.1. MALDI-TOF MS 

Matrix-assisted laser desorption/ionization time-of-flight mass spectrometry (MALDI-TOF MS) is a powerful analytical technique for identifying and characterizing biomolecules, including proteins, peptides, and microorganisms. This method has revolutionized the field of microbiology by providing rapid, accurate, and cost-effective identification of a wide range of pathogens, including bacteria, fungi, and viruses [40]. It recognizes the pathogen even in blood cultures, and it involves two main processes: matrix-assisted laser desorption/ionization (MALDI) and time-of-flight mass spectrometry (TOF MS). MALDI-TOF MS can rapidly identify bacterial species by comparing the mass spectra of unknown samples to reference in a database. It can identify specific proteins or enzymes linked to antibiotic resistance, such as beta-lactamases, helping to guide appropriate antibiotic therapy and pathogenicity mechanisms of microorganisms against antimicrobials. The advantage of MALDI-TOF MS is that it provides results within minutes to hours, significantly faster than traditional culture-based methods. It has high accuracy in species identification due to comprehensive reference databases and can identify a wide range of microorganisms and detect antibiotic resistance mechanisms. The limitations of this method are that it depends on the quality and comprehensiveness of the reference database and requires careful sample preparation to ensure reliable results. Also, this method has limited detection for some pathogens. Nevertheless, MALDI-TOF MS has become an invaluable tool in clinical microbiology, offering rapid and accurate identification of a broad spectrum of pathogens, particularly in the case of *H. pylori* bacteria. As technology and databases continue to improve, the scope and precision of MALDI-TOF MS in microbial diagnostics are expected to expand further.

A recent research study was made to compare MALDI-TOF MS with FT-IR. Isolates were obtained from children with severe infections who were admitted to Alder Hey Children’s Hospital as part of a multicenter study. As a result, MALDI-TOF MS turned out to have a 95.8% accuracy [41]. 

While MALDI-TOF MS has all these features that result in a quick turnaround of identification, the main limitation arises in resource-limited settings where the initial cost of setting the equipment is a major problem that is under discussion [42]. 

### 4.2. Biosensor Technology 

Biosensor technology represents a significant advancement in diagnosing *H. pylori* infection, offering rapid, sensitive, and non-invasive alternatives to traditional diagnostic methods. As technology continues to evolve, biosensors have the potential to become a standard tool in the clinical management of *H. pylori* and other infectious diseases, improving patient outcomes, and streamlining diagnostic workflows. A biosensor is an analytical device that combines a biological recognition element with a physicochemical transducer to detect and quantify specific biological molecules. There are different types of biosensors for the diagnosis of *H. pylori* infection. 

#### 4.2.1. Electrochemical Biosensors

These biosensors measure changes in electrical properties (current, voltage, or impedance) due to the interaction between the biological recognition element and the target analyte—detecting *H. pylori*-specific antigens or urease activity. For instance, urease produced by *H. pylori* hydrolyzes urea to produce ammonia, which can be detected electrochemically [43].

#### 4.2.2. Optical Biosensors

These sensors detect changes in optical properties (absorbance, fluorescence, or surface plasmon resonance) upon interaction with the target analyte. This detection of *H. pylori* DNA or protein markers uses labeled antibodies or nucleic acid probes that produce a detectable optical signal [44]. 

#### 4.2.3. Piezoelectric Biosensors

These sensors measure changes in mass or mechanical properties on the sensor surface due to the binding of the target analyte—detection of *H. pylori* antigens or antibodies occurs with the use of quartz crystal microbalance (QCM) technology.

#### 4.2.4. Thermal Biosensors

These sensors detect changes in temperature as a result of biochemical reactions. They monitor the enzymatic activity of urease produced by *H. pylori*, which generates heat as a byproduct. The advantages of biosensors in diagnosis include results in real-time or within a few minutes, which is significantly faster than traditional culture or histological methods. It has high sensitivity and specificity; biosensors can detect low concentrations of target molecules with high specificity, reducing false positives and negatives. Many biosensors can be used with non-invasive samples such as breath, saliva, or urine, minimizing patient discomfort. Biosensors can be designed as portable devices, allowing for point-of-care testing and use in resource-limited settings. Biosensors reduce the need for expensive reagents and complex laboratory infrastructure. Challenges include integrating biosensor technology into existing healthcare systems, which requires addressing issues related to data management, regulatory approvals, and clinician training [45].

Ongoing research focuses on enhancing biosensors’ sensitivity, specificity, and multiplexing capabilities and developing novel recognition elements and transduction mechanisms. There have been important solutions to the limitations experienced, as discussed by one study that developed a rapid detection system combining RPA and CRISPR-Cas12a to diagnose and monitor *H. pylori* in clinical settings by targeting the UreB gene [46]. The system is highly sensitive, detecting as few as 50–100 copies, and it completes the detection process in just 40 min. This method provides a fast, sensitive, and efficient diagnostic tool, ideal for use in hospitals or testing sites with limited resources [46]. 

### 4.3. Next-Generation Sequencing (NGS)

Next-generation sequencing (NGS) has revolutionized genomics by allowing the rapid and comprehensive analysis of genetic material [47]. Unlike traditional sequencing methods like Sanger sequencing, NGS can sequence millions of fragments simultaneously, offering unprecedented speed, throughput, and accuracy. This technology has broad applications in medical research, diagnostics, personalized medicine, and many other fields.

NGS involves the following key steps: DNA or RNA samples are fragmented into smaller pieces. Adapters (short DNA sequences) are added to the ends of these fragments to create a library. The library fragments are amplified using techniques like PCR to increase the quantity of DNA. The amplified fragments are sequenced in parallel, producing millions of short reads. Advanced bioinformatics tools align these short reads to a reference genome or assemble them de novo, followed by variant calling, annotation, and interpretation. Several NGS platforms and technologies are available, each with unique features and applications. 

Illumina sequencing involves the incorporation of fluorescently labeled nucleotides, which are detected in real time. Advantages of this method include high accuracy, high throughput, and cost-effectiveness. Ion Torrent sequencing is sequencing by synthesis with the detection of hydrogen ions released during nucleotide incorporation. Its advantages include fast run times and a relatively low cost. Single-molecule real-time (SMRT) sequencing involves real-time detection of fluorescently labeled nucleotides as they are incorporated by DNA polymerase. Oxford nanopore sequencing involves DNA or RNA molecules passing through a nanopore, and changes in ionic current are measured to determine the sequence.

While costs have decreased significantly, NGS remains expensive for some applications, limiting its accessibility. Technological advancements and economies of scale are expected to reduce costs further and broaden accessibility. Variability in sample preparation, sequencing protocols, and data analysis can impact results. Currently, companies are working on measures to reduce the cost and have been successful in this regard with costs decreasing from $5400 to $200 over the last 10 years [48]. 

Lastly, handling sensitive genetic information raises ethical and privacy issues. Policies and frameworks are needed to protect patient privacy and ensure the ethical use of genetic data [49]. 

## 5. Importance of Advanced Diagnostics in Treatment Strategies

The newer diagnostic techniques have a promising role to play in the case of antibiotic resistance, which is a significant concern for refractory *H. pylori*. Early methods for detecting antibiotic resistance in *H. pylori*, such as PCR-restriction fragment length polymorphism (RFLP) and real-time PCR, focused on identifying specific mutations in a small target gene region, requiring prior knowledge of these mutations [50]. This approach limited the discovery of novel or complex resistance mechanisms. NGS methods, including Illumina and PacBio sequencing, enable whole genome sequencing (WGS), which offers a complete view of bacterial genotypes and the ability to track rare or complex antibiotic resistance mechanisms. WGS is particularly useful in areas with high resistance rates, though challenges remain, such as low bacterial DNA content in gastric biopsies and discrepancies between genotypic and phenotypic resistance [50]. While traditional antimicrobial susceptibility testing methods are well-standardized and detect phenotypic resistance, they require high bacterial concentrations. They can be influenced by observer variability, failing to detect heteroresistance. Despite its advantages, NGS needs to be better understood and standardized for routine use in diagnostic microbiology laboratories [50]. MALDI-TOF MS is also being used to detect enzymatic mutations responsible for antibiotic resistance in this species, specifically, beta lactamases and rRNA methyltransferases [51]. 

Although our diagnostic and therapeutic modalities to combat *H. pylori* have increased, there are some barriers to the implementation in resource-limited settings with a high prevalence. Social determinants of health create social divisions based on socio-economic and socio-political groups, leading to health disparities. Addressing social determinants in healthcare, such as racial and ethnic inequalities and language barriers, can improve patient education, treatment compliance, and follow-up care. Understanding these factors helps healthcare providers and policymakers develop initiatives to enhance healthcare outcomes. Socioeconomic status significantly influences access to healthcare, especially in the United States, where lower status is often linked to poorer health outcomes due to limited access to care [52]. Addressing chronic diseases associated with aging becomes increasingly essential as the global population ages. Expanding health insurance and reducing racial, ethnic, and financial disparities are essential to improving future healthcare access and outcomes [52]. 

## 6. Conclusions

*H. pylori* infection continues to pose a significant global health challenge, with its prevalence influenced by various factors, including geography, socioeconomic status, and healthcare quality. While non-invasive diagnostic methods like serological assays, stool antigen tests, and urea breath tests offer accessible and effective tools for identifying H. pylori, their utility is often complemented by invasive techniques such as histopathological examination, bacterial culture, and molecular diagnostics. These approaches, particularly with recent advancements like MALDI-TOF MS, enable precise characterization of the bacterium, aiding in targeted treatment strategies, especially in antibiotic resistance. Advances in diagnostic technologies and improvements in healthcare infrastructure are crucial in managing and reducing the burden of *H. pylori*-related diseases. Addressing the disparities in infection rates and ensuring timely diagnosis and treatment are essential for mitigating the impact of *H. pylori* and preventing its associated complications, thereby improving global health outcomes. Further research should discuss the role of targeting socioeconomic determinants of health, for example, insurance expansion, widespread production of newer advances, and collaboration with primary care, to reduce the gap of economic disparities between resource-limited and resource-abundant settings. 

## References

[1] Burucoa C., Axon A. (2017). Epidemiology of *Helicobacter pylori* infection. Helicobacter.

[2] Mentis A., Lehours P., Mégraud F. (2015). Epidemiology and Diagnosis of *Helicobacter pylori* infection. Helicobacter.

[3] Azarkar Z., Jafarnejad M., Sharifzadeh G. (2011). The relationship between helicobacter pylori infection and myocardial infarction. Caspian J. Intern. Med..

[4] Hatakeyama M. (2004). Oncogenic mechanisms of the *Helicobacter pylori* CagA protein. Nat. Rev. Cancer.

[5] Cover T.L., Blanke S.R. (2005). *Helicobacter pylori* VacA, a paradigm for toxin multifunctionality. Nat. Rev. Microbiol..

[6] Peek R.M., Blaser M.J. (2022). *Helicobacter pylori* and gastrointestinal tract adenocarcinomas. Nat. Rev. Cancer.

[7] Salama N.R., Hartung M.L., Müller A. (2013). Life in the human stomach: Persistence strategies of the bacterial pathogen *Helicobacter pylori*. Nat. Rev. Microbiol..

[8] Amieva M., Peek R.M. (2016). Pathobiology of *Helicobacter pylori*–Induced Gastric Cancer. Gastroenterology.

[9] Yokota K., Osaki T., Hayashi S., Yokota S.I., Takeuchi H., Rimbara E., Ojima H., Sato T., Yonezawa H., Shibayama K. (2022). Establishment of a reference panel of *Helicobacter pylori* strains for antimicrobial susceptibility testing. Helicobacter.

[10] National Cancer Institute Helicobacter pylori (*H. pylori*) and Cancer. https://www.cancer.gov/about-cancer/causes-prevention/risk/infectious-agents/h-pylori-fact-sheet#:~:text=However%2C%20infection%20with%20H.,esophagus%20and%20gastroesophageal%20reflux%20disease.

[11] Baj J., Forma A., Sitarz M., Portincasa P., Garruti G., Krasowska D., Maciejewski R. (2020). *Helicobacter pylori* Virulence Factors-Mechanisms of Bacterial Pathogenicity in the Gastric Microenvironment. Cells.

[12] Charitos I.A., D’Agostino D., Topi S., Bottalico L. (2021). 40 Years of *Helicobacter pylori*: A Revolution in Biomedical Thought. Gastroenterol. Insights.

[13] Parente J.M., da Silva B.B., Palha-Dias M.P., Zaterka S., Nishimura N.F., Zeitune J.M. (2006). *Helicobacter pylori* infection in children of low and high socioeconomic status in northeastern Brazil. Am. Trop. Med. Hyg..

[14] Carroll I.M., Ahmed N., Beesley S.M., Khan A.A., Ghousunnissa S., Moráin C.A.Ó., Habibullah C.M., Smyth C.J. (2004). Microevolution between paired antral and paired antrum and corpus *H. pylori* isolates recovered from individual patients. J. Med. Microbiol..

[15] Marques S.B. Prevalence of *H. pylori* Infection Associated with Clinical Disorders Diagnosed by Upper Gastrointestinal Endoscopies, Retrospective Analysis of 1478 Cases. https://www.teses.usp.br/teses/disponiveis/5/5147/tde-19022010-151204/en.php.

[16] Kayali S., Aloe R., Bonaguri C., Gaiani F., Manfredi M., Leandro G., Fornaroli F., Di Mario F., De’ Angelis G.L. (2018). Non-invasive tests for the diagnosis of helicobacter pylori: State of the art. Acta Biomed..

[17] Chey W.D., Wong B.C.Y. (2007). American College of Gastroenterology Guideline on the Management of *H. pylori* Infection. Am. J. Gastroenterol..

[18] Kusters J.G., van Vliet A.H., Kuipers E.J. (2006). Pathogenesis of *H. pylori* infection. Clin. Microbiol. Rev..

[19] Gisbert J.P., Pajares J.M. (2004). Stool antigen test for the diagnosis of *H. pylori* infection: A systematic review. Helicobacter.

[20] Kazemi S., Tavakkoli H., Habizadeh M.R., Emami M.H. (2011). Diagnostic values of *H. pylori* diagnostic tests: Stool antigen test, urea breath test, rapid urease test, serology and histology. J. Res. Med. Sci..

[21] Peng N.J., Lai K.H., Lo G.H., Hsu P.I. (2009). Comparison of noninvasive diagnostic tests for *H. pylori* infection. Med. Princ. Pract..

[22] Gatta L., Perna F., Ricci C., Osborn J.F., Tampieri A., Bernabucci V., Miglioli M., Vaira D. (2004). A rapid immunochromatographic assay for *H. pylori* in stool before and after treatment. Aliment. Pharmacol. Ther..

[23] Imrie C., Rowland M., Bourke B., Drumm B. (2001). Limitations to carbonl3-labeled urea breath testing for *H. pylori* in infants. J. Pediatr..

[24] Ferwana M., Abdulmajeed I., Alhajiahmed A., Madani W., Firwana B., Hasan R., Altayar O., Limburg P.J., Murad M.H., Knawy B. (2015). Accuracy of urea breath test in *Helicobacter pylori* infection: Meta-analysis. World J. Gastroenterol..

[25] Ricci C., Holton J., Vaira D. (2007). Diagnosis of *Helicobacter pylori*: Invasive and Non-Invasive Tests. Best. Pract. Res. Clin. Gastroenterol..

[26] Best L.M.J., Takwoingi Y., Siddique S., Selladurai A., Gandhi A., Low B., Yaghoobi M., Gurusamy K.S. (2018). Non-Invasive Diagnostic Tests for *Helicobacter pylori* Infection. Cochrane Database Syst. Rev..

[27] Lee H.S. Histopathologic Diagnosis of *H. pylori* Infection and Associated Gastric Diseases. https://snucm.elsevierpure.com/en/publications/histopathologic-diagnosis-of-h-pylori-infection-and-associated-ga.

[28] Yadav R., Sagar M. (2022). Comparison of Different Histological Staining Methods for Detection of *Helicobacter pylori* Infection in Gastric Biopsy. Cureus.

[29] Dixon M.F., Genta R.M., Yardley J.H., Correa P., the Participants in the International Workshop on the Histopathology of Gastritis, Houston 1994 (1996). Classification and Grading of Gastritis: The Updated Sydney System. Am. J. Surg. Pathol..

[30] Mărginean C.O., Meliț L.E., Săsăran M.O. (2022). Traditional and Modern Diagnostic Approaches in Diagnosing Pediatric Helicobacter Pylori Infection. Children.

[31] Patel S.K., Pratap C.B., Jain A.K., Gulati A.K., Nath G. (2014). Diagnosis of *Helicobacter pylori*: What Should Be the Gold Standard?. World J. Gastroenterol..

[32] Hirschl A.M., Makristathis A. (2007). Methods to Detect *Helicobacter pylori*: From Culture to Molecular Biology. Helicobacter.

[33] Bénéjat L., Ducournau A., Lehours P., Mégraud F. (2018). Real-Time PCR for *Helicobacter pylori* Diagnosis. The Best Tools Available. Helicobacter.

[34] Gantuya B., El Serag H.B., Saruuljavkhlan B., Azzaya D., Matsumoto T., Uchida T., Oyuntsetseg K., Oyunbileg N., Davaadorj D., Yamaoka Y. (2021). Advantage of 16S RRNA Amplicon Sequencing in *Helicobacter pylori* Diagnosis. Helicobacter.

[35] Zhu F., Zhang X., Li P., Zhu Y. (2023). Effect of *Helicobacter pylori* Eradication on Gastric Precancerous Lesions: A Systematic Review and Meta-Analysis. Helicobacter.

[36] Trung T.T., Minh T.A., Anh N.T. (2019). Value of CIM, CLO Test and Multiplex PCR for the Diagnosis of Helicobacter Pylori Infection Status in Patients with Gastritis and Gastric Ulcer. Asian Pac. J. Cancer Prev..

[37] Valenzuela G., Dickinson D. (2009). Sensitivity of Gastric Biopsy and Stool Antigen for *H. pylori* Diagnosis: 98. Am. J. Gastroenterol..

[38] Liao E., Yu C., Lai J., Lin C., Chen C., Chang W., Chien D. (2023). A pilot study of non-invasive diagnostic tools to detect *Helicobacter pylori* infection and peptic ulcer disease. Sci. Rep..

[39] Qiu E., Li Z., Han S. (2021). Methods for detection of *Helicobacter pylori*, current options and developments. Braz. J. Microbiol..

[40] Cardos A.I., Maghiar A., Zaha D.C., Pop O., Fritea L., Miere Groza F., Cavalu S. (2022). Evolution of Diagnostic Methods for *Helicobacter pylori* Infections: From Traditional Tests to High Technology, Advanced Sensitivity and Discrimination Tools. Diagnostics.

[41] Evangelista A.J., Ferreira T.L. (2022). Matrix-assisted laser desorption/ionization time-of-flight mass spectrometry in the diagnosis of microorganisms. Future Microbiol..

[42] Singhal N., Kumar M., Kanaujia P.K., Virdi J.S. (2015). MALDI-TOF mass spectrometry: An emerging technology for microbial identification and diagnosis. Front. Microbiol..

[43] Zhang Y., Wang S., Hu B., Zhao F., Xiang P., Ji D., Chen F., Liu X., Yang F., Wu Y. (2016). Direct Detection of Helicobacter Pylori in Biopsy Specimens using a High-Throughput Multiple Genetic Detection System. Future Microbiol..

[44] Herrera-Domínguez M., Morales-Luna G., Mahlknecht J., Cheng Q., Aguilar-Hernández I., Ornelas-Soto N. (2023). Optical Biosensors and Their Applications for the Detection of Water Pollutants. Biosensors.

[45] Zheng Y., Song X., Fredj Z., Bian S., Sawan M. (2023). Challenges and perspectives of multi-virus biosensing techniques: A review. Anal. Chim. Acta..

[46] Liu H., Wang J., Hu X., Tang X., Zhang C. (2023). A rapid and high-throughput *Helicobacter pylori* RPA-CRISPR/Cas12a-based nucleic acid detection system. Clin. Chim. Acta.

[47] Bocu R. (2024). Extended Review Concerning the Integration of Electrochemical Biosensors into Modern IoT and Wearable Devices. Biosensors.

[48] Illumina. Cost of NGS. https://www.illumina.com/science/technology/next-generation-sequencing/beginners/ngs-cost.html.

[49] Pohl D., Keller P.M., Bordier V., Wagner K. (2019). Review of current diagnostic methods and advances in *Helicobacter pylori* diagnostics in the era of next generation sequencing. World J. Gastroenterol..

[50] Saracino I.M., Pavoni M., Zullo A., Fiorini G., Lazzarotto T., Borghi C., Vaira D. (2021). Next Generation Sequencing for the Prediction of the Antibiotic Resistance in *Helicobacter pylori*: A Literature Review. Antibiotics.

[51] Hrabák J., Chudácková E., Walková R. (2013). Matrix-assisted laser desorption ionization-time of flight (maldi-tof) mass spectrometry for detection of antibiotic resistance mechanisms: From research to routine diagnosis. Clin. Microbiol. Rev..

[52] Rammohan R., Magam S.G., Joy M., Natt D., Patel A., Tadikonda A., Desai J., Bunting S., Yost R.M., Akande O. (2023). Unpacking the Racial Gap: *Helicobacter pylori* Infection Clearance Among Different Racial Groups. Cureus.

